# Meta-Analysis of the Relationship between Common Type 2 Diabetes Risk Gene Variants with Gestational Diabetes Mellitus

**DOI:** 10.1371/journal.pone.0045882

**Published:** 2012-09-24

**Authors:** Hongyan Mao, Qin Li, Shujun Gao

**Affiliations:** 1 Department of Gynecology and Obstetrics, Qidong People's Hospital, Jiangsu, People's Republic of China; 2 Gynecology and Obstetrics Hospital, Fudan University, Shanghai, People's Republic of China; Wadsworth Center, United States of America

## Abstract

**Background:**

A number of case-control studies were conducted to investigate the association of common type 2 diabetes (T2D) risk gene polymorphisms with gestational diabetes mellitus (GDM). However, these studies have yielded contradictory results. We therefore performed a meta-analysis to derive a more precise estimation of the association between these polymorphisms and GDM, hence achieve a better understanding to the relationship between T2D and GDM.

**Methods:**

PubMed, EMBASE, ISI web of science and the Chinese National Knowledge Infrastructure databases were systematically searched to identify relevant studies. Data were abstracted independently by two reviewers. A meta-analysis was performed to examine the association between 9 polymorphisms from 8 genes and susceptibility to GDM. Odds ratios (ORs) and 95% confidence intervals (95% CIs) were calculated. Heterogeneity among articles and their publication bias were also tested.

**Results:**

We identified 22 eligible studies including a total of 10,336 GDM cases and 17,445 controls. We found 8 genetic polymorphisms were significantly associated with GDM in a random-effects meta-analysis. These polymorphisms were in or near the following genes: TCF7L2 (rs7903146), MTNR1B (rs10830963), IGF2BP2 (rs4402960), KCNJ11 (rs5219), CDKAL1 (rs7754840), KCNQ1 (rs2237892 and rs2237895) and GCK (rs4607517); while no association was found for PPARG with GDM risk. Similar results were also observed under dominant genetic model for these polymorphisms.

**Conclusions:**

This meta-analysis found 8 genetic variants associated with GDM. The relative contribution and relevance of the identified genes in the pathogenesis of GDM should be the focus of future studies.

## Introduction

Gestational diabetes mellitus (GDM) is defined as glucose intolerance that is first detected during pregnancy [1]. It is characterized by impaired insulin secretion and action [2], [3]. Gestational diabetes complicates about 1–3% of all pregnancies in the western world [4], whereas 5–10% among of Asian women [5]. Although its exact etiology is unknown, accumulating evidence recognizes GDM as a quintessential multifactorial disease in which environmental triggers interact with genetic variants [6]. Given the fact that women with a history of GDM are at an increased risk of developing type 2 diabetes (T2D) later in their lives [7] and women with a family history of diabetes may be predisposed to an increased risk of GDM [8], it is plausible to hypothesize that GMD may share the same risk factors and genetic susceptibilities with T2D. However, knowledge regarding the genetics of GDM is very limited so far [9].

Recently, spectacular advance was made in identifying susceptible genes involved in T2D through genome-wide association strategy (GWAS) [10], [11]. Consequently, a number of novel genetic variants (*PPARG*, *KCNJ11*, *IGF2BP2*, *KCNQ1*, *TCF7L2*, *CDKAL1*, and *MTNR1B*) were shown to increase the risk of T2D in reproducible studies. Therefore, several studies have examined the association of these newly identified loci using a candidate gene approach for GDM. It has been reported that the pathophysiological changes of GDM are similar to those observed in T2D, which is characterized by peripheral insulin resistance accompanied by an insulin secretory defect [12], [13]. Functional studies showed that these new diabetogenic genes took part in many steps of the process, for instance, impaired β-cell function (*CDKAL1*, *IGF2BP2*, *KCNQ1*, *KCNJ11, MTNR1B*), insulin resistance (*PPARG*, *TCF7L2*), and abnormal utilization of glucose (*GCK*) [14]–[23].

Genetic association studies can be problematic to reproduce due to inadequate statistical power, multiple hypothesis testing, population stratification, publication bias, and phenotypic heterogeneity. Considering the lack of sufficient evidence about the effect of candidate genes of T2D on GDM and the conflicting results reported, we therefore performed a meta-analysis to assess the association between the most commonly studied polymorphisms in the *PPARG*, *CDKAL1*, *KCNQ1*, *IGF2BP2*, *TCF7L2*, *KCNJ11*, *MTNR1B, GCK* genes and GDM risk.

## Materials and Methods

### Literature search strategy

Genetic association studies polymorphism and GDM risk published up to April, 2012 were identified through systematic searches in PubMed, EMBASE, ISI Web of Science and the Chinese National Knowledge Infrastructure (CNKI) databases. No language restrictions were applied. The search strategy consisted of multiple queries combining: ‘gestational diabetes mellitus’ and ‘variations’ or ‘polymorphisms’. In addition, the names of specific genes and polymorphisms were combined with the topic ‘gestational diabetes mellitus’. All reference lists from the main reports and relevant reviews were hand searched for additional eligible studies.

### Eligible studies and data extraction

Eligible studies had to meet all the following criteria: (1) original papers containing independent data, (2) identification of gestational diabetes mellitus cases was confirmed pathologically, (3) case–control or cohort studies and (4) genotype distribution information in cases and controls or odds ratio (OR) with its 95% confidence interval (CI) and P-value. The major reasons for exclusion of studies were (1) overlapping data; (2) case-only studies, family based studies, and review articles.

Data extraction was performed independently by two reviewers. Review reports from the two were than compared to identify any inconsistency, and differences were resolved by further discussion among all authors. For each included study, the following information was extracted from each report according to a fixed protocol: first author's surname, publication year, definition and numbers of cases and controls, frequency of genotypes, Hardy–Weinberg equilibrium status, source of controls, mean age of cases and controls, body mass index (BMI), ethnicity, and genotyping method.

### Statistical methods

The strength of association between the genetic polymorphism and GDM was accessed by calculating odds ratio (OR) with 95% confidence interval (CI). For single nucleotide polymorphisms (SNPs), the frequency of the risk allele was compared between diabetic cases and non-diabetic controls. Additional pooled estimates were also given with corresponding results under dominant genetic model.

Cochran's chi-square-based Q statistic test was performed in order to assess possible heterogeneity between the individual studies and thus to ensure that each group of studies was suitable for meta-analysis [24]. ORs were pooled according to the method of DerSimonian and Laird that takes into account the variation between studies, and 95% CI were constructed using Woolf's method [25], [26]. The Z test was used to determine the significance of the pooled OR. Pre-specified stratified analyses were performed to explain heterogeneity or investigate whether the reported association was present in a subgroup. Stratified analysis was performed for ethnicity (Caucasian vs East Asian origin).

Funnel plots was used to provide diagnosis of the potential publication bias. Egger's regression test was also conducted to identify small study effects [27]. Chi-square test was used to check if there was significant deviation from Hardy–Weinberg equilibrium (HWE) among the control subjects in each study. All statistical analyses were carried out with the Stata software version 10.0 (Stata Corporation, College Station, TX). The type I error rate was set at 0.05. All the p-values were for two-sided analysis.

## Results

### Characteristics of studies

**Figure 1:**
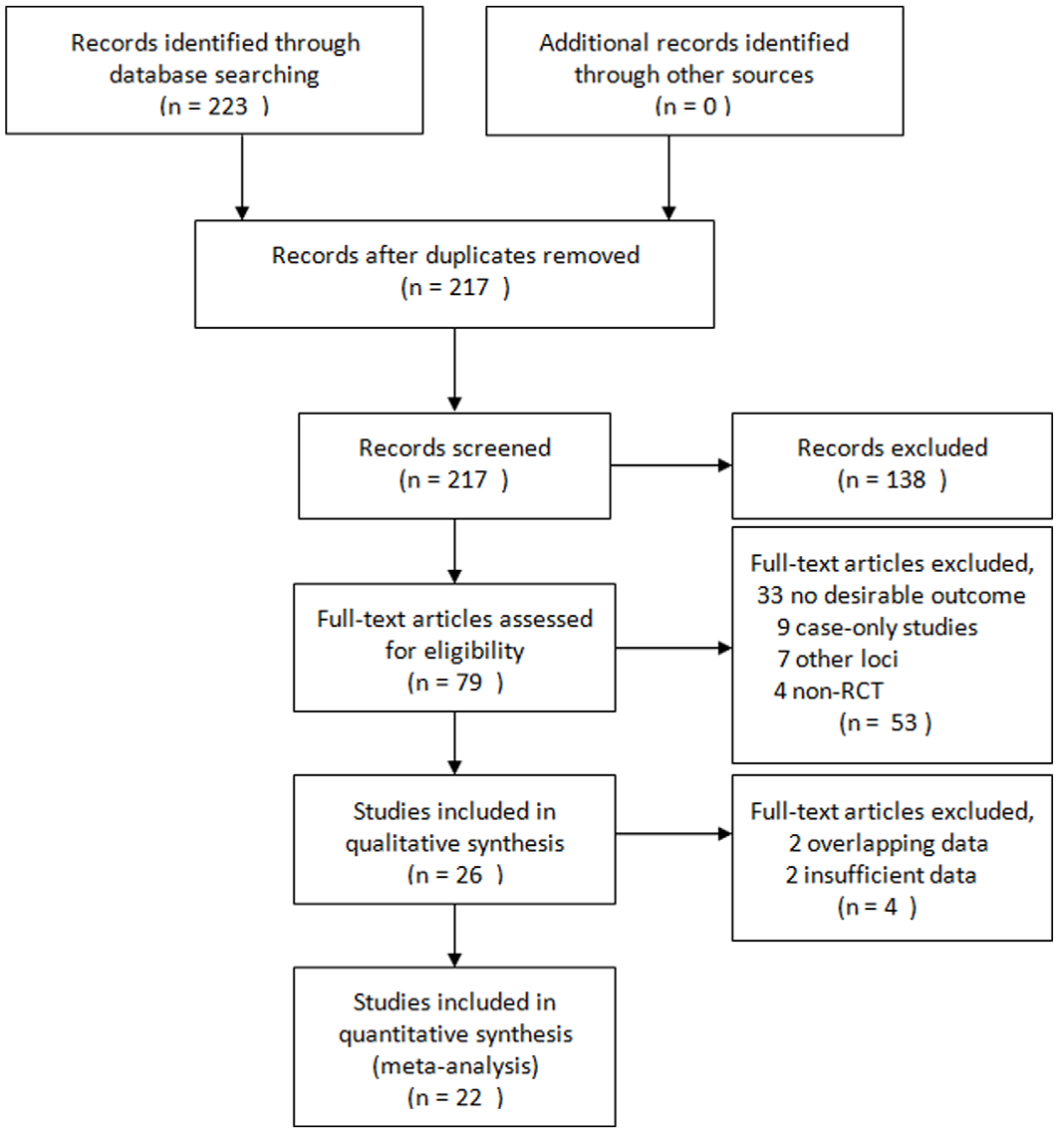
The flow chart of the included studies.

In all, we included 22 studies in this meta-analysis, with a total of 10,336 cases and 17,445 controls concerning 9 genetic variants in or near 8 genes. The detailed characteristics of the studies included were shown in Table 1. The study selection process is shown in Figure 1. These polymorphisms were found to occur in frequencies consistent with Hardy–Weinberg equilibrium in the control populations of the vast majority of the published studies. Details of analyses of all assessed genetic variants are provided in Table 2.

**Table 1:** Characteristics of the studies included in the meta-analysis.

Study	Year	Ethnicity	Case	Control	No. of case/control	Genotyping method	Age of case/control	BMI of case/control
Kwak [28]	2012	Korean	OGTT confirmed	Normal fasting glucose	1399/2025	Affymetrix array, TaqMan	32.1/62.5	24.2/24.4
Kim [29]	2011	Korean	OGTT confirmed	Normal glucose tolerant	908/966	TaqMan	33.2/32.2	23.3/21.4
Deng [30]	2011	Chinese	GDM patients	Non-diabetic participants	87/91	Sequencing	31.8/29.7	23.6/21.5
Heude [31]	2011	French	OGTT confirmed	Normal glucose tolerant	109/1571	TaqMan	NA/NA	NA/NA
Wang [32]	2011	Chinese	OGTT confirmed	Normal glucose tolerant	705/1029	TaqMan	32.0/30.0	21.7/21.5
Pappa [33]	2011	Greek	GDM per IGDW criteria	Normal glucose tolerant	148/107	RFLP	32.5/26.7	26.0/24.3
Papadopoulou [34]	2011	Swede	OGTT confirmed	Normal glucose tolerant	803/1110	TaqMan	NA/NA	NA/NA
Shin [35]	2010	Korean	OGTT confirmed	Healthy	930/628	TaqMan	33.2/NA	23.3/NA
Cheng [36]	2010	Chinese	OGTT confirmed	Normal glucose tolerant	55/173	RFLP	27.0/29.6	NA/NA
Kwak [37]	2010	Korean	OGTT confirmed	Normal fasting glucose	869/632	TaqMan	27.9/64.7	23.1/23.3
Santos [38]	2010	Euro-Brazilian	GDM per ADA criteria	Healthy	150/600	RFLP	31.7/24.9	33.5/24.8
Zhou [39]	2009	Chinese	OGTT confirmed	Normal glucose tolerant	520/916	RFLP	32.5/30.7	21.4/21.4
Cho [40]	2009	Korean	OGTT confirmed	Normal fasting glucose	868/632	TaqMan	32.0/64.7	23.1/23.3
Zhu [41]	2009	Chinese	GDM patients	Non-diabetic participants	179/180	RFLP	28.1/27.4	24.6/23.4
Lauenborg [14]	2008	Dane	OGTT confirmed	Normal glucose tolerant	276/2383	TaqMan	43.1/45.2	28.9/25.0
Shaat [42]	2007	Swede	OGTT confirmed	Non-diabetic participants	637/1232	TaqMan	32.3/30.5	NA/NA
Tok [43]	2006	Turkish	OGTT confirmed	Normal glucose tolerant	100/62	RFLP	33.5/31.6	26.6/23.0
Shaat [44]	2006	Swede	OGTT confirmed	Non-diabetic participants	641/1229	RFLP	NA/NA	NA/NA
Shaat [45]	2005	Swede	OGTT confirmed	Normal glucose tolerant	588/1180	TaqMan	32.2/30.5	NA/NA
Shaat [46]	2004	Swede	OGTT confirmed	Normal glucose tolerant	500/550	RFLP	32.3/NA	30.1/NA
Chiu [47]	1994	American	OGTT confirmed	Non-diabetic participants	174/99	SSCP	28.3/22.1	33.9/22.5
Unpublished data	/	Chinese	OGTT confirmed	Normal glucose tolerant	50/50	RFLP	30.5/28.8	31.0/27.9

OGTT: Oral Glucose Tolerance Test, IGDW: International Gestational Diabetes Workshop, ADA: American Diabetes Association, NA: Not Available.

**Table 2:** Results of the pooled data analyses for the 9 studied polymorphisms and gestational diabetes mellitus risk.

Variants per gene	Risk allele	Total/subgroup	No. data sets	No. of case/control	Risk allele			Dominant model		
					OR (95%CI)	P(Z)	P(Q)	OR (95%CI)	P(Z)	P(Q)
PPARG rs1801282	C	Total	11	2908/6940	1.01 (0.96–1.06)	0.80	1.00	1.14 (0.68–1.91)	0.63	0.45
		Caucasian	5	1559/5721	1.00 (0.94–1.06)	0.98	0.99	1.01 (0.58–1.76)	0.98	0.44
		East Asian	4	1149/1035	1.02 (0.93–1.11)	0.70	0.99	2.43 (0.38–15.43)	0.35	0.27
TCF7L2 rs7903146	T	Total	6	3148/6550	1.51 (1.39–1.65)	<10^−5^	0.77	1.69 (1.51–1.89)	<10^−5^	0.51
		Caucasian	4	1812/4681	1.51 (1.38–1.65)	<10^−5^	0.48	1.71 (1.49–1.96)	<10^−5^	0.28
		East Asian	2	1336/1869	1.55 (1.16–2.09)	0.004	0.90	1.56 (1.24–2.22)	0.001	0.75
MTNR1B rs10830963	G	Total	5	3094/4111	1.34 (1.18–1.52)	<10^−5^	0.02	1.46 (1.25–1.72)	<10^−5^	0.11
IGF2BP2 rs4402960	T	Total	4	2304/5228	1.21 (1.08–1.36)	0.001	0.09	1.25 (1.07–1.49)	0.003	0.06
		East Asian	3	2030/2894	1.24 (1.07–1.44)	0.004	0.07	1.27 (1.12–1.43)	0.0002	0.81
KCNJ11 rs5219	T	Total	5	2305/5569	1.15 (1.06–1.24)	0.0004	0.99	1.25 (1.10–1.42)	0.001	0.88
		Caucasian	3	991/3698	1.17 (1.05–1.30)	0.005	0.98	1.25 (1.07–1.46)	0.006	0.72
		East Asian	2	1314/1871	1.13 (1.02–1.26)	0.03	0.93	1.11 (1.03–1.20)	0.02	0.29
CDKAL1 rs7754840	C	Total	4	2959/3675	1.43 (1.20–1.71)	<10^−4^	0.0003	1.51 (1.33–1.82)	<10^−4^	0.008
KCNQ1 rs2237892	C	Total	3	2285/2168	1.20 (1.09–1.31)	<10^−4^	0.70	1.42 (1.18–1.71)	0.0002	0.97
KCNQ1 rs2237895	C	Total	3	2286/2168	1.20 (1.09–1.31)	0.0001	0.75	1.31 (1.16–1.48)	<10^−4^	0.54
GCK rs4607517	A	Total	5	2135/4193	1.12 (1.02–1.23)	0.01	0.41	1.15 (1.01–1.30)	0.04	0.43

### Genetic variants involved in β-cell function

Six genetic variants in five genes thought to be related to β-cell function were reproducibly associated with GDM. rs4402960 of *IGF2BP2* was associated with GDM (OR = 1.21, 95% CI = 1.08–1.36, *P* = 0.001; Supplementary figure 1) in four studies (n = 8,732), also in the subgroup among East Asian diabetes mellitus patients (OR = 1.24, 95% CI = 1.07–1.44, *P* = 0.004). The rs10830963 in *MTNR1B* were studied in five studies concerning diabetic patients form East Asian population. The overall OR of the G allele for GDM was 1.34 (95% CI = 1.18–1.52, *P*<10^−5^; Supplementary figure 2). *CDKAL1* rs7754840 polymorphism was studied in four studies and was associated with GDM in the meta-analysis (OR = 1.43, 95% CI = 1.20–1.71, *P*<10^−4^; Supplementary figure 3). All four studies contained diabetic patients of East Asian descent. For *KCNJ11* rs5219, our meta-analysis gave an overall OR of 1.15 (95% CI = 1.06–1.24, *P* = 0.0004; Supplementary figure 4) without statistically significant between-study heterogeneity. Significantly increased GDM risks were also found for Caucasian and East Asian populations when stratified by ethnicity. Significantly increased GDM risks were found for rs2237892 and rs2237895 of *KCNQ1* with per-allele OR of 1.20 (95% CI = 1.09–1.31, *P*<10^−4^; Supplementary figure 5) and 1.20 (95% CI = 1.09–1.31, *P* = 0.0001; Supplementary figure 6) respectively. In addition, statistically significant results were also observed for these polymorphisms under dominant genetic model. After adjusting for multiple testing using Bonferroni correction, all significant associations for these polymorphisms under the allelic comparison and dominant genetic model remained.

### Genetic variants involved in insulin resistance

A variant in *PPARG*, rs1801282, was the most studied polymorphism in GDM, with 11 data sets resulting in a pooled odds ratio of 1.01 (95% CI = 0.96–1.06, *P* = 0.80, Supplementary figure 7). Subsidiary analyses of ethnicity yielded a per-allele OR for East Asians of 1.02 (95% CI: 0.93–1.11, *P* = 0.70) and for Caucasians of 1.00 (95% CI: 0.94–1.06, *P* = 0.98). Similar results were also detected under dominant genetic model.

rs7903146 of *TCF7L2*, which is an important component in Wnt signaling pathway involved in development of the pancreas and islets, was associated with GDM (C allele: OR = 1.51, 95% CI = 1.39–1.65, *P*<10^−5^, Supplementary figure 8; dominant model: OR = 1.69, 95% CI = 1.51–1.89, *P*<10^−5^). Stratification by ethnicity indicated that the polymorphism was significantly associated with GDM for East Asians and Caucasians in all genetic models. After Bonferroni correction, significant associations still maintained for the polymorphism.

### Genetic variants involved in glucose utilization

rs4607517 of *GCK*, which is first rate-limiting step in the glycolysis pathway, was significantly associated with GDM in the meta-analysis (OR = 1.12, 95% CI = 1.02–1.23, *P* = 0.01, Supplementary figure 9). Similar results were also found using dominant genetic model with OR of 1.15 (95% CI = 1.01–1.30, *P* = 0.04). However, for GCK rs4607517 association was no longer statistically significant using dominant genetic model after Bonferroni correction.

### Publication bias

Begger's funnel plot and Egger's test were used to identify the potential publication biases of the literature, the shapes of the funnel plots appeared to be symmetrical (Supplementary figure 10–18) for polymorphisms in *PPARG, TCF7L2, MTNR1B, IGF2BP2, KCNJ11, CDKAL1, KCNQ1 and GCK*, suggesting that there was no obvious publication bias. Egger's test was used to provide further statistical evidence; similarly, the results showed no significant publication bias in this meta-analysis for these polymorphisms (*P*>0.05 for all polymorphisms).

## Discussion

Large sample and unbiased epidemiological studies of predisposition genes polymorphisms could provide insight into the in vivo relationship between candidate genes and complex diseases. In this meta-analysis, 8 genetic variants were found to be associated with increased GDM susceptibility. Genetic studies of several T2D associated variants in relation to GDM has been performed previously, but this is the first complete overview assessing for these genetic variants that are reproducibly associated with the presence of GDM. This information could lead to improved insight into underlying pathogenetic mechanisms and the relationship between GDM and T2D. These results support a role for the following in the pathogenesis of GDM: impaired β-cell function, insulin resistance and abnormal utilization of glucose. During pregnancy, women are faced with increased adiposity and increased insulin resistance. The insulin resistance that develops during pregnancy is explained in part by the increased production of human placental lactogen, estrogen, and prolactin [48]–[50]. Those who have limited β-cell capacity for the compensation of insulin resistance are likely to develop GDM [9]. Women with GDM are assumed to have decreased β-cell insulin secretory function similar to T2D [9]. After parturition, nearly one-half of these women progress to T2D within 5 years [51]–[53]. Therefore, GDM is often regarded as a herald of type 2 diabetes in later life. Functional studies remain to be performed to establish the precise roles of these variants and pathways.

The identification of GDM susceptibility variants can lead to novel biological insights and improved measures of individual etiological processes, as indicated previously [54]. Individual etiological processes could allow preventive and therapeutic interventions in complex disease to be tailored to individuals on the basis of their genetic profiles. From prediction studies with genetic variants for T2D, it has been shown that 20 established genetic variants in T2D have an AUC of 0.54 (0.5 means no predictive value, 1.0 is perfect prediction), in contrast to the Framingham offspring and Cambridge risk scores (AUC of 0.78 and 0.72, respectively). Interestingly, addition of genetic information to phenotype-based risk models did not improve prediction [55]. It is also possible that for GDM the genotypic risk does not exceed the risk contributed by conventional risk factors (e.g. BMI, age, term of pregnancies), which means that the predictive value of risk variants for GDM would be limited [56]. Although genetic prediction and use of personalized medicine in GDM remains a new undertaking, prediction is likely to improve as additional disease variants are detected and replicated [57].

Novel biological insights may lead to development of new therapeutic targets, biomarkers and opportunities for disease prevention. Hypothesis-free approaches, such as GWAS, are most promising in this respect. At present, it seems wise to focus on assessing the relevance of previously detected genetic variants. As common SNPs associated with GDM and detected by GWAS may represent rare genetic variants with large effects, sequencing the regions surrounding highly significant and replicated genomic regions to detect rare variants appears to be reasonable. Follow-up in vitro and in vivo studies could then assess the functional relevance of these variants in GDM.

In interpreting the results, some limitations of this meta-analysis should be addressed. Publication bias is a concern in all meta-analyses even though the use of a statistical test did not show it. Negative studies are less likely to be published, potentially leading to an overestimation of effects. Moreover, non-significant genetic associations might have been underreported in published articles. Therefore, the effect estimates of the present study should be interpreted with caution, especially in cases where associations were based on small numbers of studies and/or small sample numbers. Second, in the subgroup analysis by ethnicity, the number of studies and subjects analyzed was small, and the statistical power was so low that caution should be taken in interpreting these results. Finally, the overall outcomes were based on individual unadjusted ORs, while a more precise evaluation should be adjusted by other potentially suspected factors including age, BMI, and environmental factors.

To the best of our knowledge, this study was the first comprehensive meta-analysis to assess the relationship between the T2D related gene polymorphisms and GDM susceptibility. Our meta-analysis identified 8 genetic variants associated with GDM. As studies among other populations are currently limited, further studies including a wider spectrum of subjects should be carried to investigate the role of those variants in other populations, which should lead to better, comprehensive understanding of the association between the genetic polymorphism and GDM. For future studies, gene–gene and gene–environment interactions should also be considered.

## Supporting Information

Figure S1 **Meta-analysis of the association between** ***IGF2BP2*** **rs4402960 polymorphism and the risk for gestational diabetes mellitus.**
(TIF)

Figure S2 **Meta-analysis of the association between** ***MTNR1B*** **rs10830963 polymorphism and the risk for gestational diabetes mellitus.**
(TIF)

Figure S3 **Meta-analysis of the association between** ***CDKAL1*** **rs7754840 polymorphism and the risk for gestational diabetes mellitus.**
(TIF)

Figure S4 **Meta-analysis of the association between** ***KCNJ11*** **rs5219 polymorphism and the risk for gestational diabetes mellitus.**
(TIF)

Figure S5 **Meta-analysis of the association between** ***KCNQ1*** **rs2237892 polymorphism and the risk for gestational diabetes mellitus.**
(TIF)

Figure S6 **Meta-analysis of the association between** ***KCNQ1*** **rs2237895 polymorphism and the risk for gestational diabetes mellitus.**
(TIF)

Figure S7 **Meta-analysis of the association between** ***PPARG*** **rs1801282 polymorphism and the risk for gestational diabetes mellitus.**
(TIF)

Figure S8 **Meta-analysis of the association between** ***TCF7L2*** **rs7903146 polymorphism and the risk for gestational diabetes mellitus.**
(TIF)

Figure S9 **Meta-analysis of the association between** ***GCK*** **rs4607517 polymorphism and the risk for gestational diabetes mellitus.**
(TIF)

Figure S10 **Begg's funnel plot of** ***PPARG*** **rs1801282 polymorphism and gestational diabetes mellitus risk (Egger test, P = 0.15).**
(TIFF)

Figure S11 **Begg's funnel plot of** ***TCF7L2*** **rs7903146 polymorphism and gestational diabetes mellitus risk (Egger test, P = 0.18).**
(TIFF)

Figure S12 **Begg's funnel plot of** ***MTNR1B*** **rs10830963 polymorphism and gestational diabetes mellitus risk (Egger test, P = 0.69).**
(TIFF)

Figure S13 **Begg's funnel plot of** ***IGF2BP2*** **rs4402960 polymorphism and gestational diabetes mellitus risk (Egger test, P = 0.70).**
(TIFF)

Figure S14 **Begg's funnel plot of** ***KCNJ11*** **rs5219 polymorphism and gestational diabetes mellitus risk (Egger test, P = 0.76).**
(TIFF)

Figure S15 **Begg's funnel plot of** ***CDKAL1*** **rs7754840 polymorphism and gestational diabetes mellitus risk (Egger test, P = 0.25).**
(TIFF)

Figure S16 **Begg's funnel plot of** ***KCNQ1*** **rs2237892 polymorphism and gestational diabetes mellitus risk (Egger test, P = 0.34).**
(TIFF)

Figure S17 **Begg's funnel plot of** ***KCNQ1*** **rs2237895 polymorphism and gestational diabetes mellitus risk (Egger test, P = 0.77).**
(TIFF)

Figure S18 **Begg's funnel plot of** ***GCK*** **rs4607517 polymorphism and gestational diabetes mellitus risk (Egger test, P = 0.65).**
(TIFF)

Checklist S19 **PRISMA 2009.**
(DOC)
