# Supplementary figures and images for: Meta-Analysis of the Relationship between Common Type 2 Diabetes Risk Gene Variants with Gestational Diabetes Mellitus
Source: PLoS One. 2012 Sep 24;7(9):e45882. doi: 10.1371/journal.pone.0045882 (PMC3454322; doi:10.1371/journal.pone.0045882)

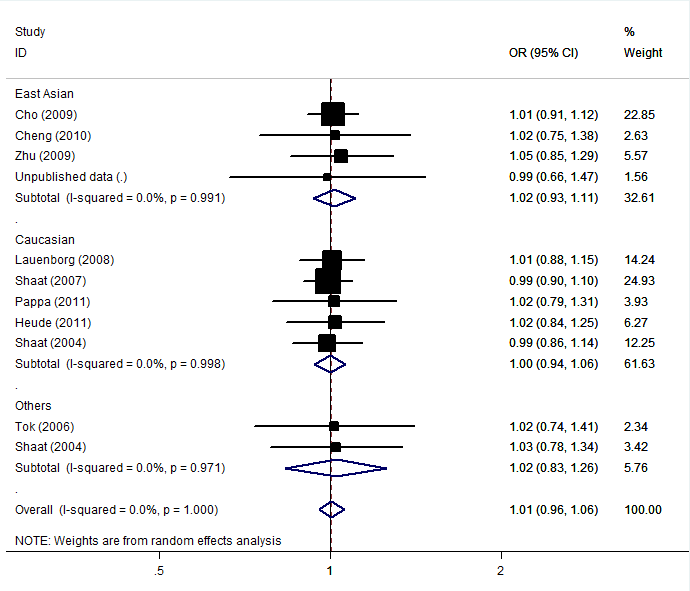

Supplement: Figure S1 — Meta-analysis of the association between IGF2BP2 rs4402960 polymorphism and the risk for gestational diabetes mellitus. (TIF) [file pone.0045882.s001.tif]

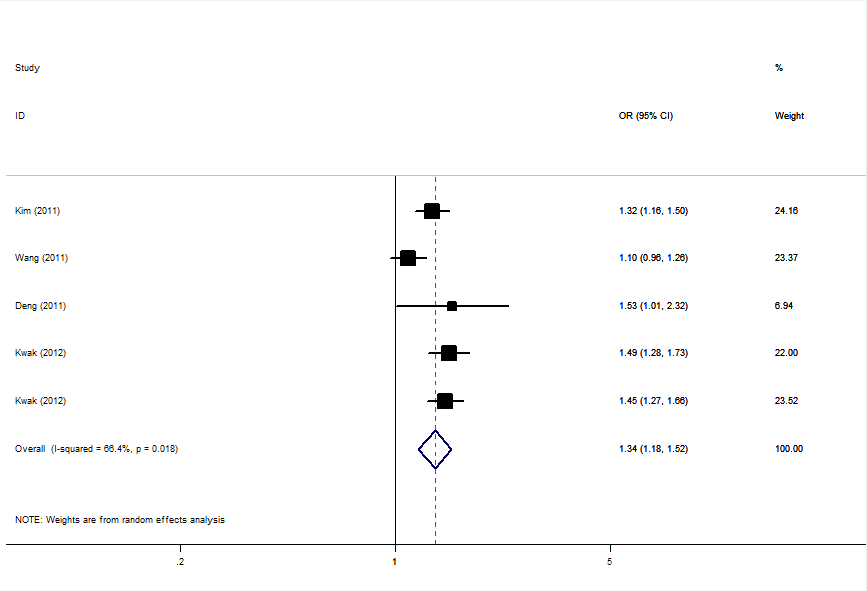

Supplement: Figure S2 — Meta-analysis of the association between MTNR1B rs10830963 polymorphism and the risk for gestational diabetes mellitus. (TIF) [file pone.0045882.s002.tif]

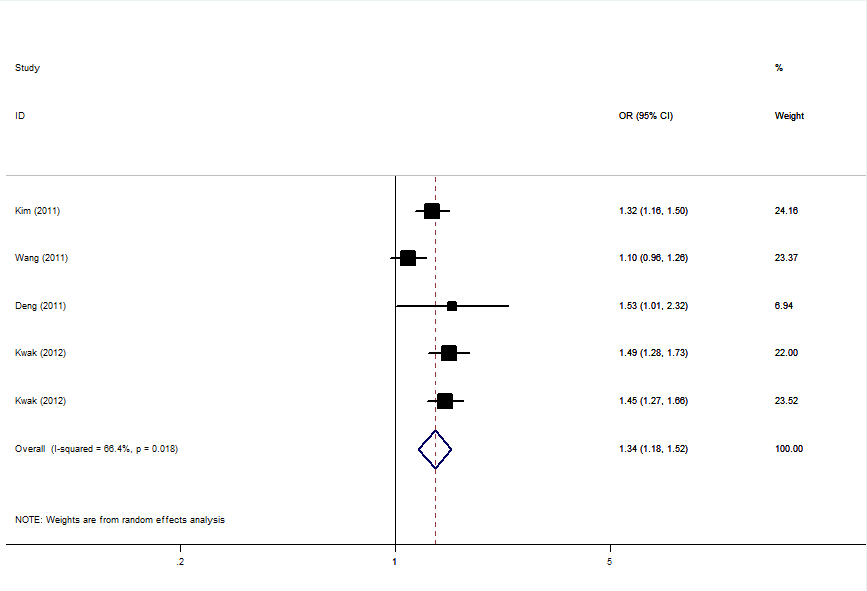

Supplement: Figure S3 — Meta-analysis of the association between CDKAL1 rs7754840 polymorphism and the risk for gestational diabetes mellitus. (TIF) [file pone.0045882.s003.tif]

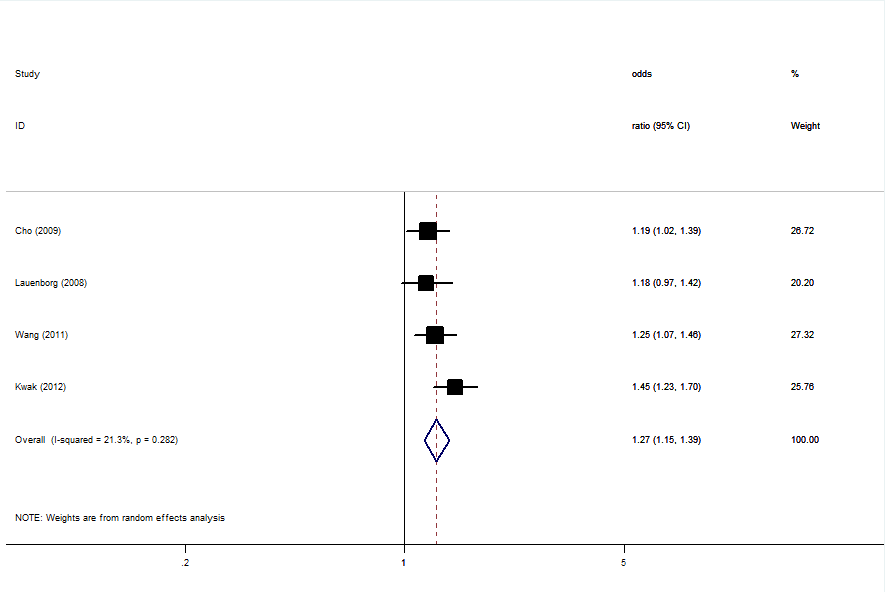

Supplement: Figure S4 — Meta-analysis of the association between KCNJ11 rs5219 polymorphism and the risk for gestational diabetes mellitus. (TIF) [file pone.0045882.s004.tif]

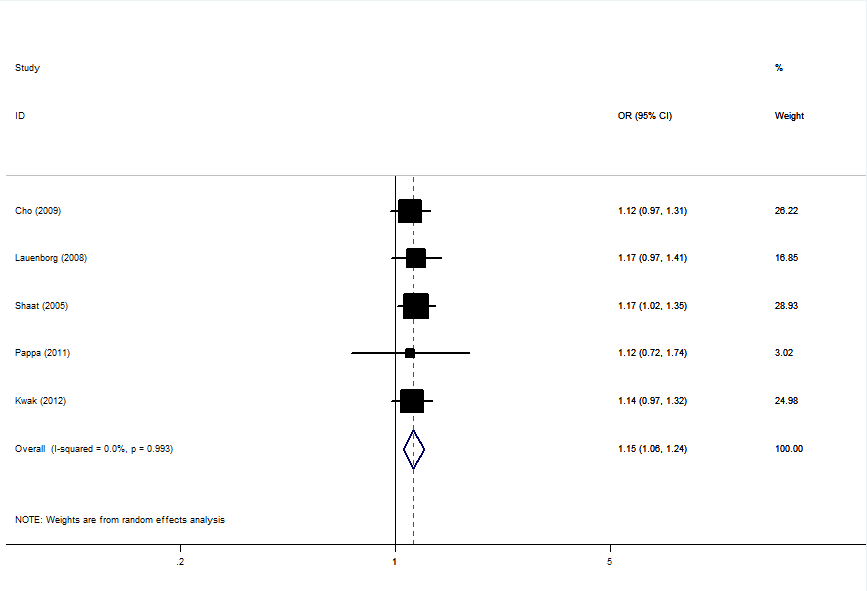

Supplement: Figure S5 — Meta-analysis of the association between KCNQ1 rs2237892 polymorphism and the risk for gestational diabetes mellitus. (TIF) [file pone.0045882.s005.tif]

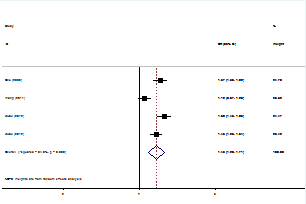

Supplement: Figure S6 — Meta-analysis of the association between KCNQ1 rs2237895 polymorphism and the risk for gestational diabetes mellitus. (TIF) [file pone.0045882.s006.tif]

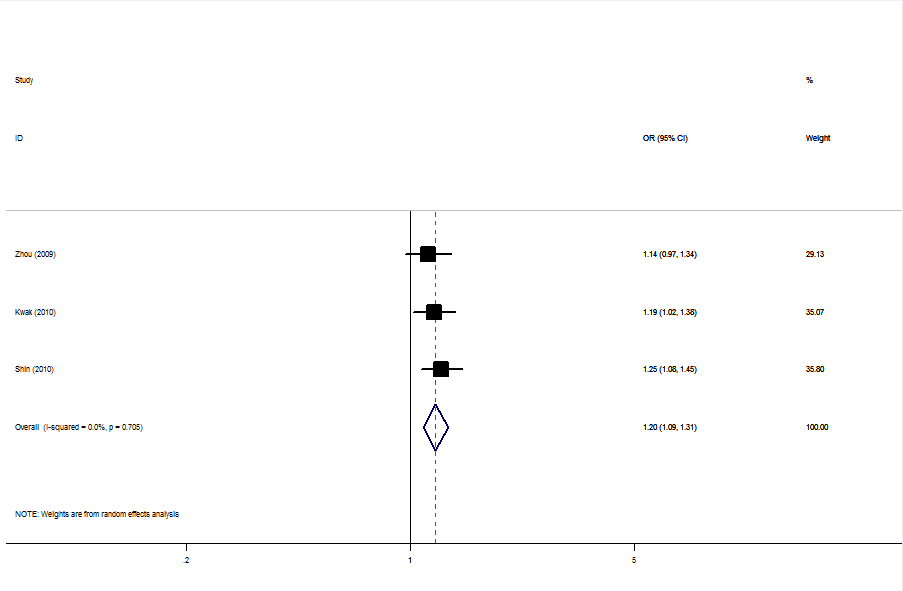

Supplement: Figure S7 — Meta-analysis of the association between PPARG rs1801282 polymorphism and the risk for gestational diabetes mellitus. (TIF) [file pone.0045882.s007.tif]

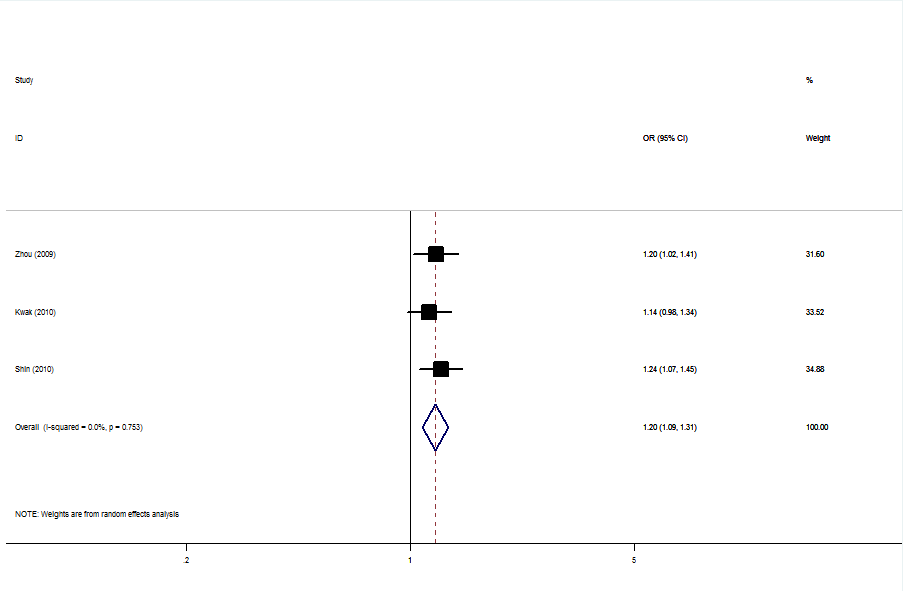

Supplement: Figure S8 — Meta-analysis of the association between TCF7L2 rs7903146 polymorphism and the risk for gestational diabetes mellitus. (TIF) [file pone.0045882.s008.tif]

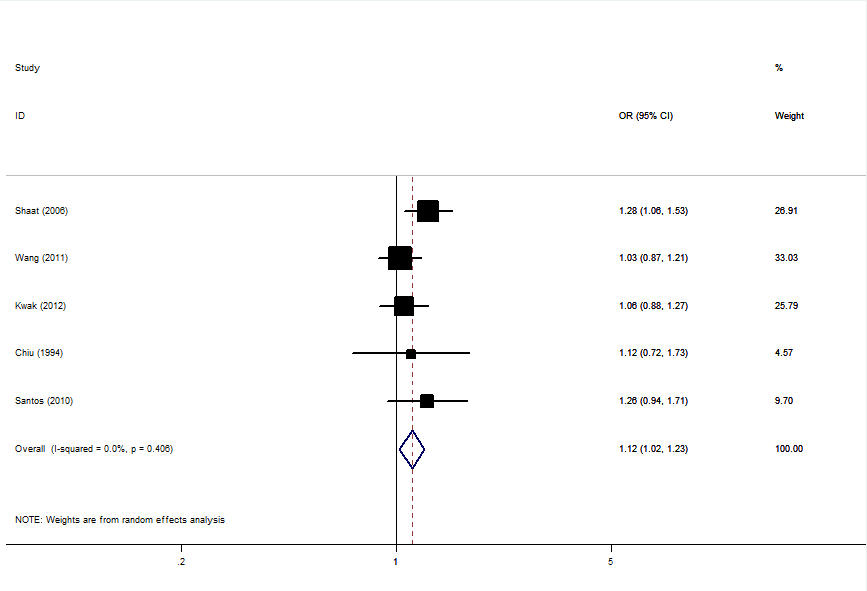

Supplement: Figure S9 — Meta-analysis of the association between GCK rs4607517 polymorphism and the risk for gestational diabetes mellitus. (TIF) [file pone.0045882.s009.tif]

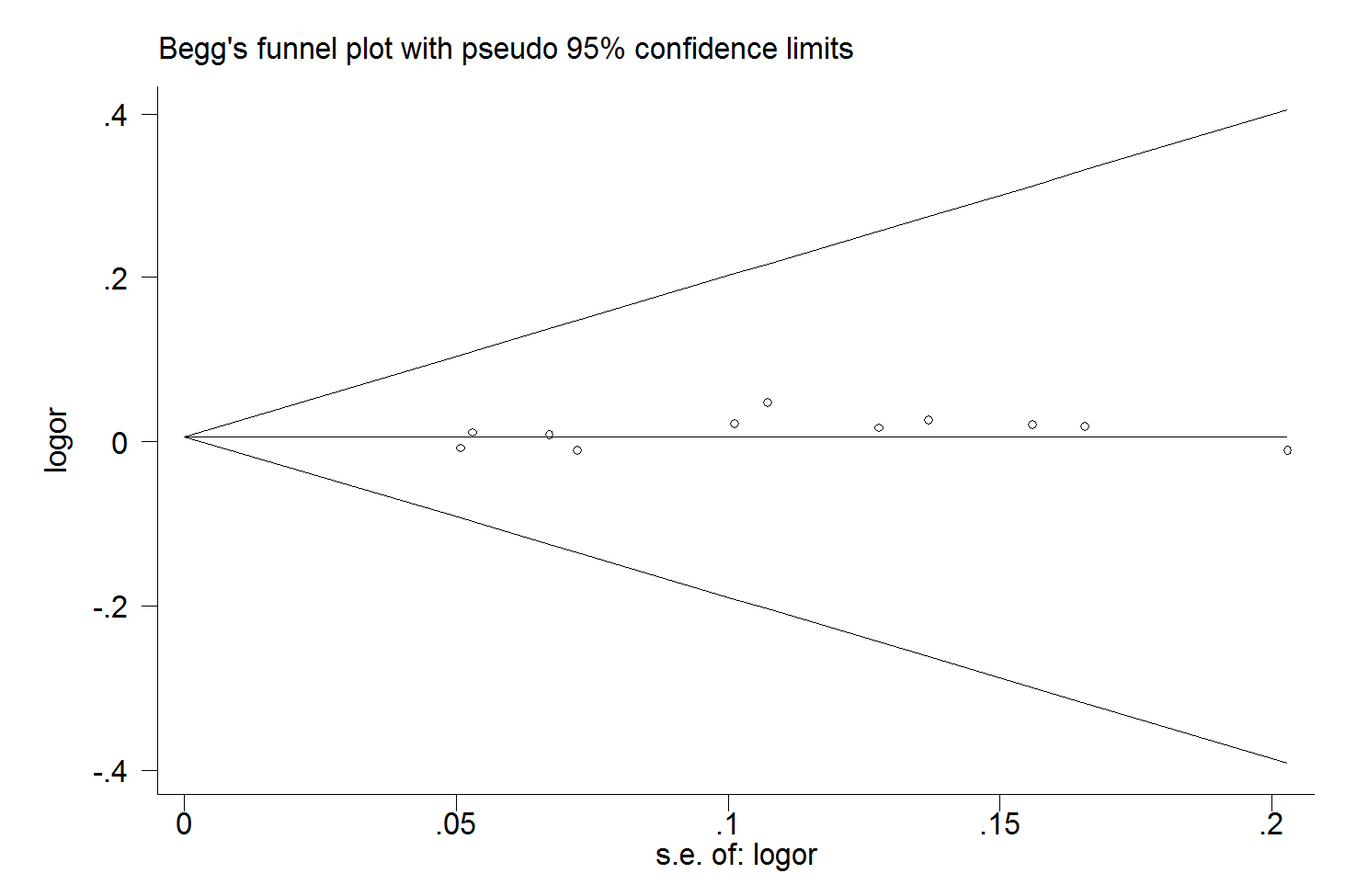

Supplement: Figure S10 — Begg's funnel plot of PPARG rs1801282 polymorphism and gestational diabetes mellitus risk (Egger test, P = 0.15). (TIFF) [file pone.0045882.s010.tif]

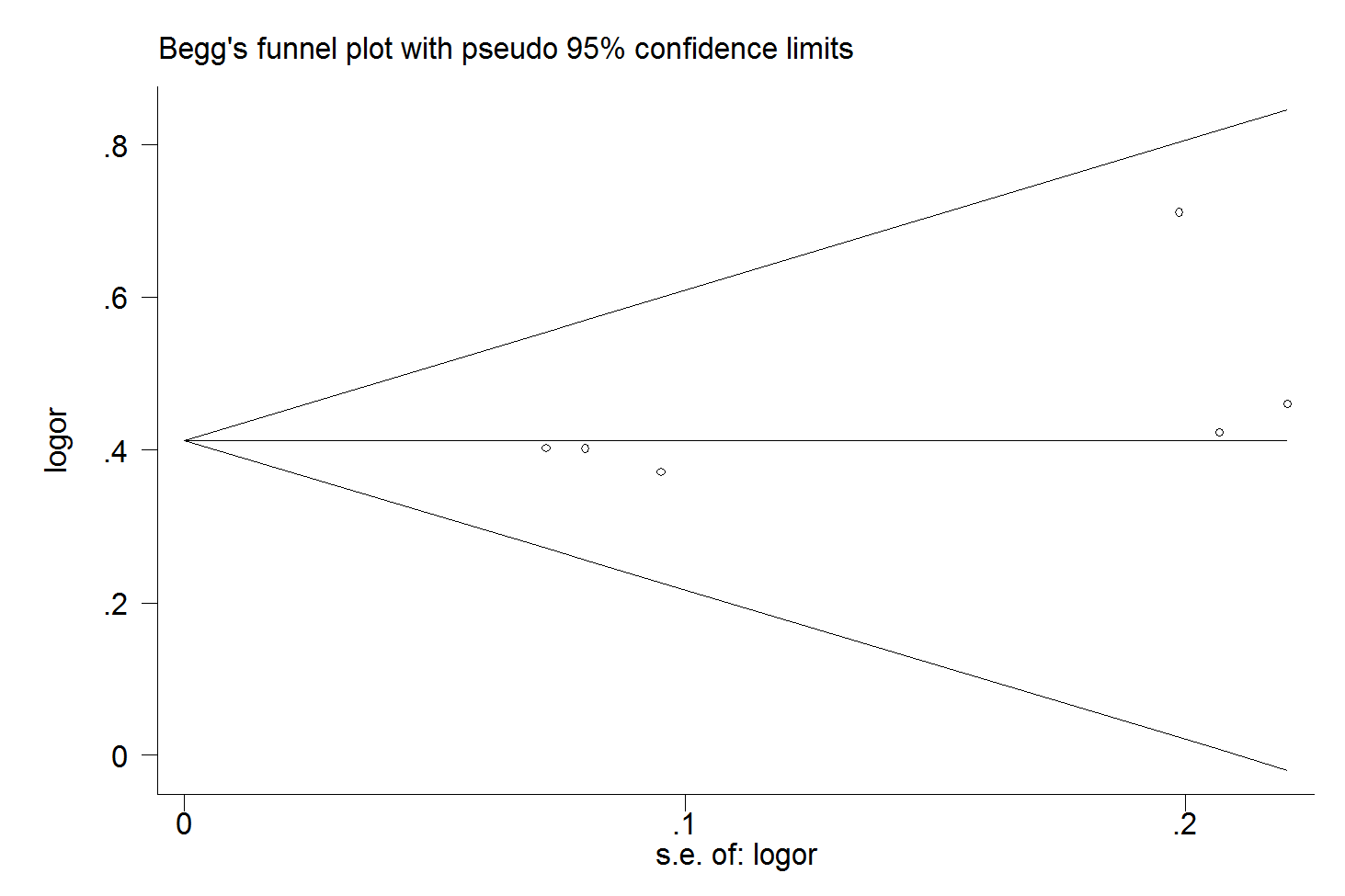

Supplement: Figure S11 — Begg's funnel plot of TCF7L2 rs7903146 polymorphism and gestational diabetes mellitus risk (Egger test, P = 0.18). (TIFF) [file pone.0045882.s011.tif]

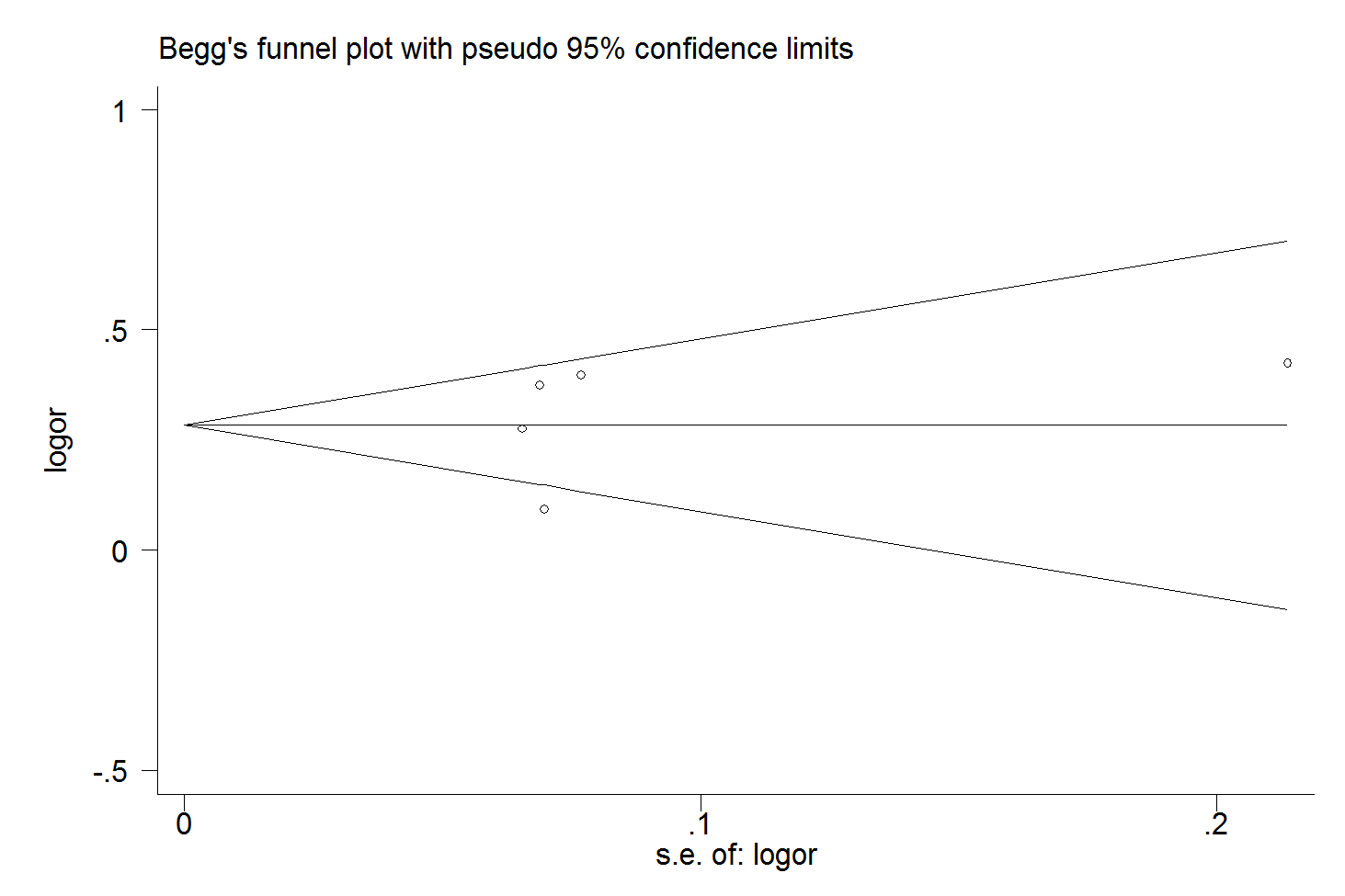

Supplement: Figure S12 — Begg's funnel plot of MTNR1B rs10830963 polymorphism and gestational diabetes mellitus risk (Egger test, P = 0.69). (TIFF) [file pone.0045882.s012.tif]

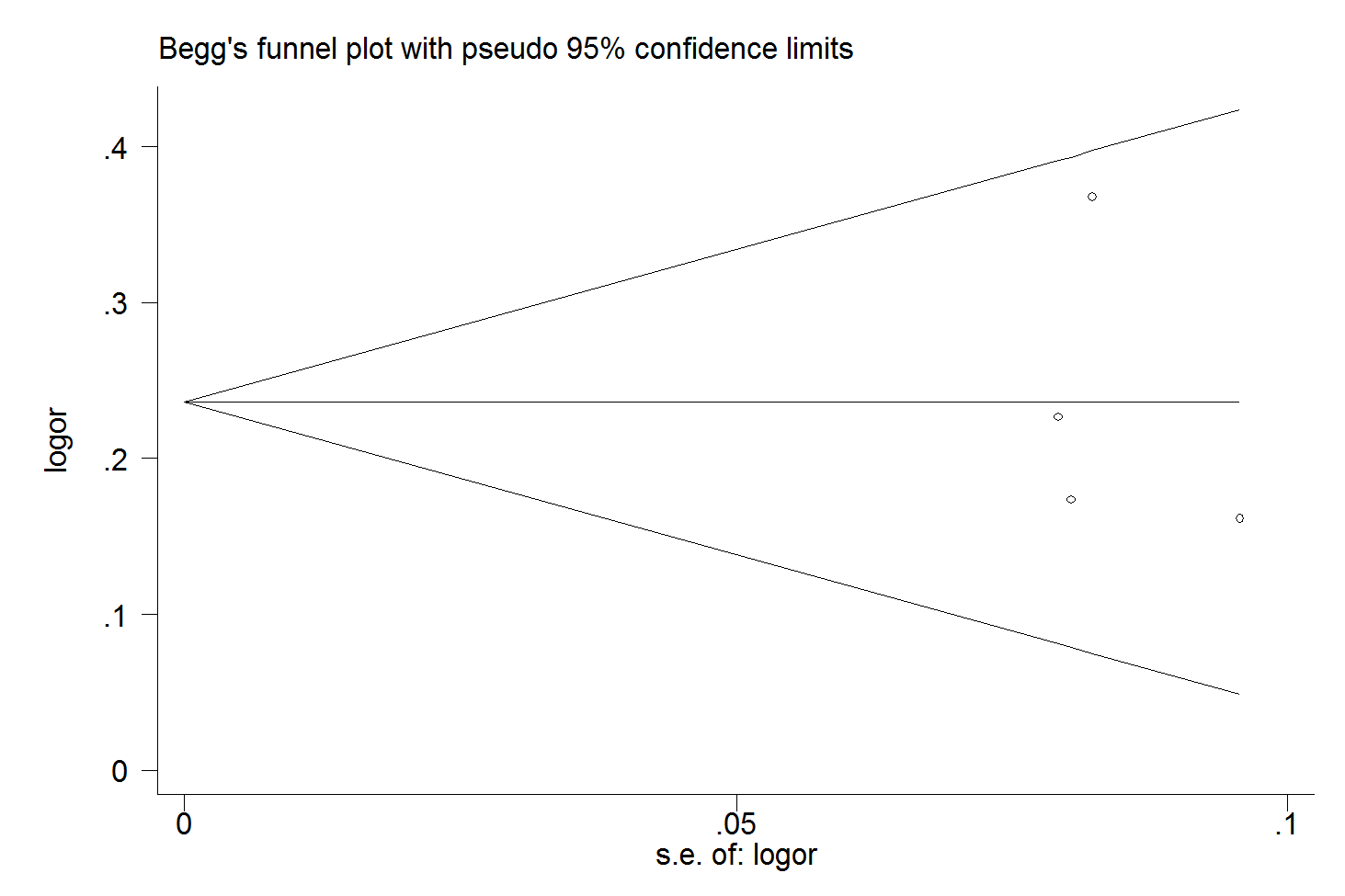

Supplement: Figure S13 — Begg's funnel plot of IGF2BP2 rs4402960 polymorphism and gestational diabetes mellitus risk (Egger test, P = 0.70). (TIFF) [file pone.0045882.s013.tif]

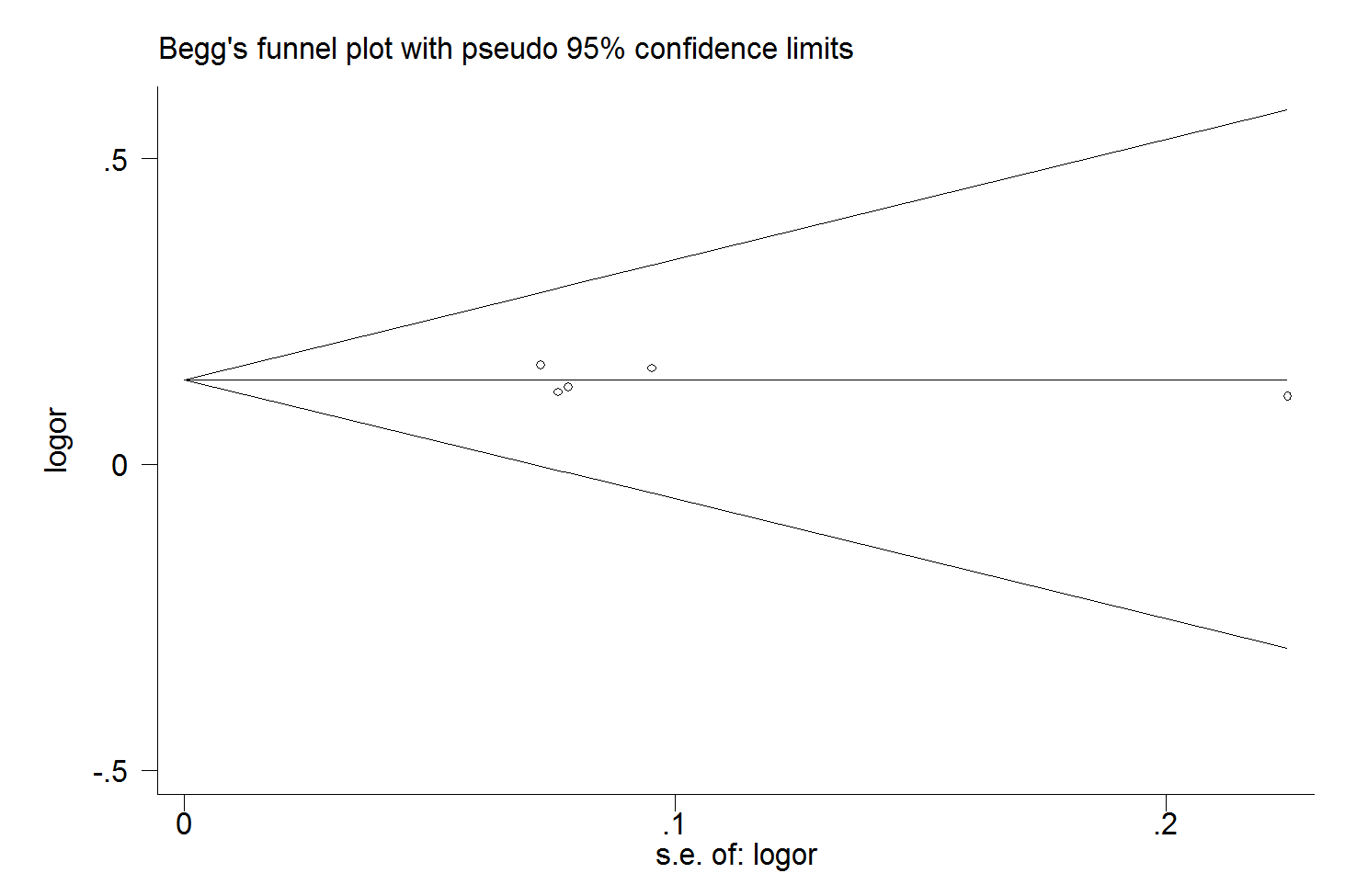

Supplement: Figure S14 — Begg's funnel plot of KCNJ11 rs5219 polymorphism and gestational diabetes mellitus risk (Egger test, P = 0.76). (TIFF) [file pone.0045882.s014.tif]

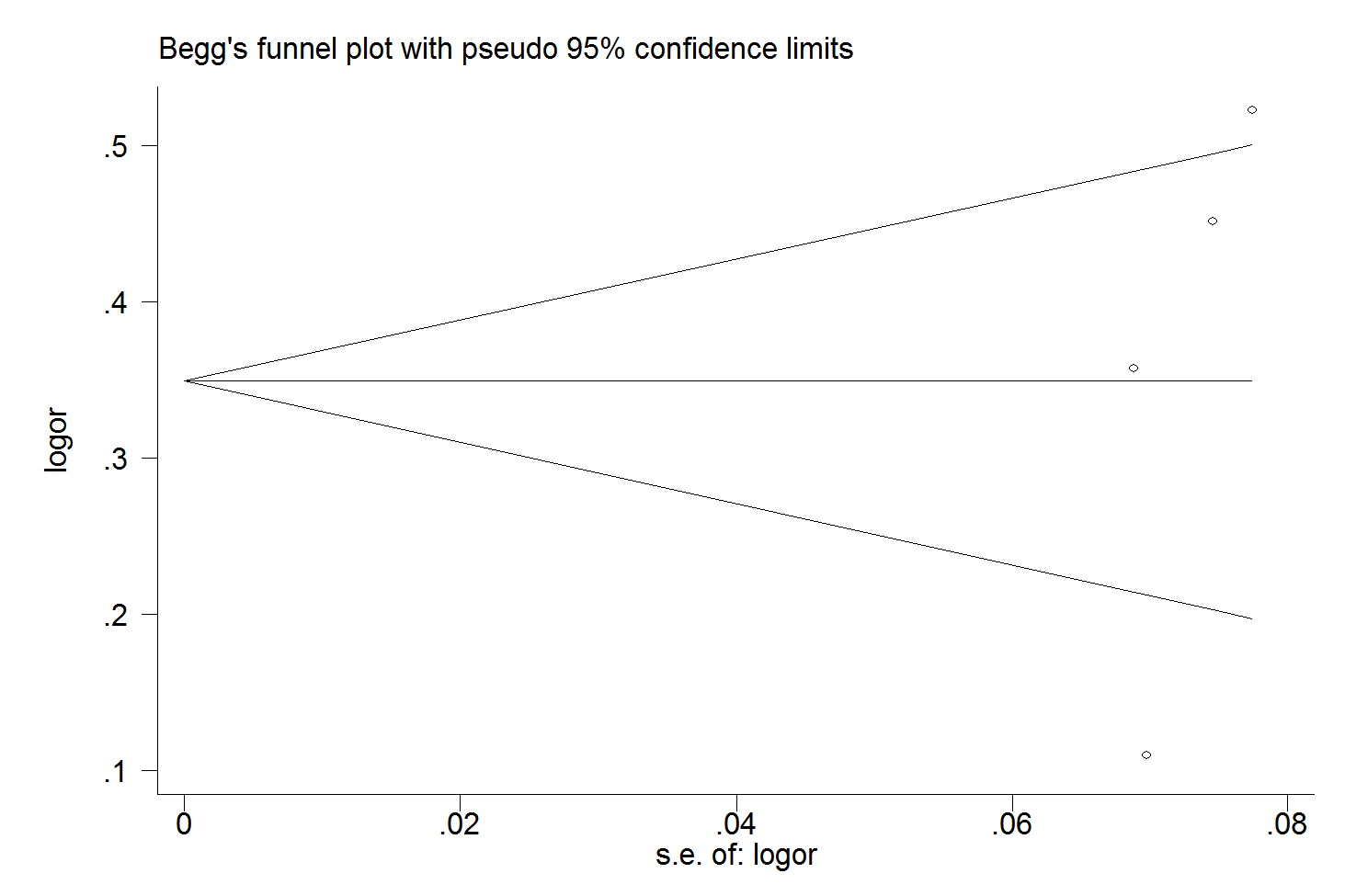

Supplement: Figure S15 — Begg's funnel plot of CDKAL1 rs7754840 polymorphism and gestational diabetes mellitus risk (Egger test, P = 0.25). (TIFF) [file pone.0045882.s015.tif]

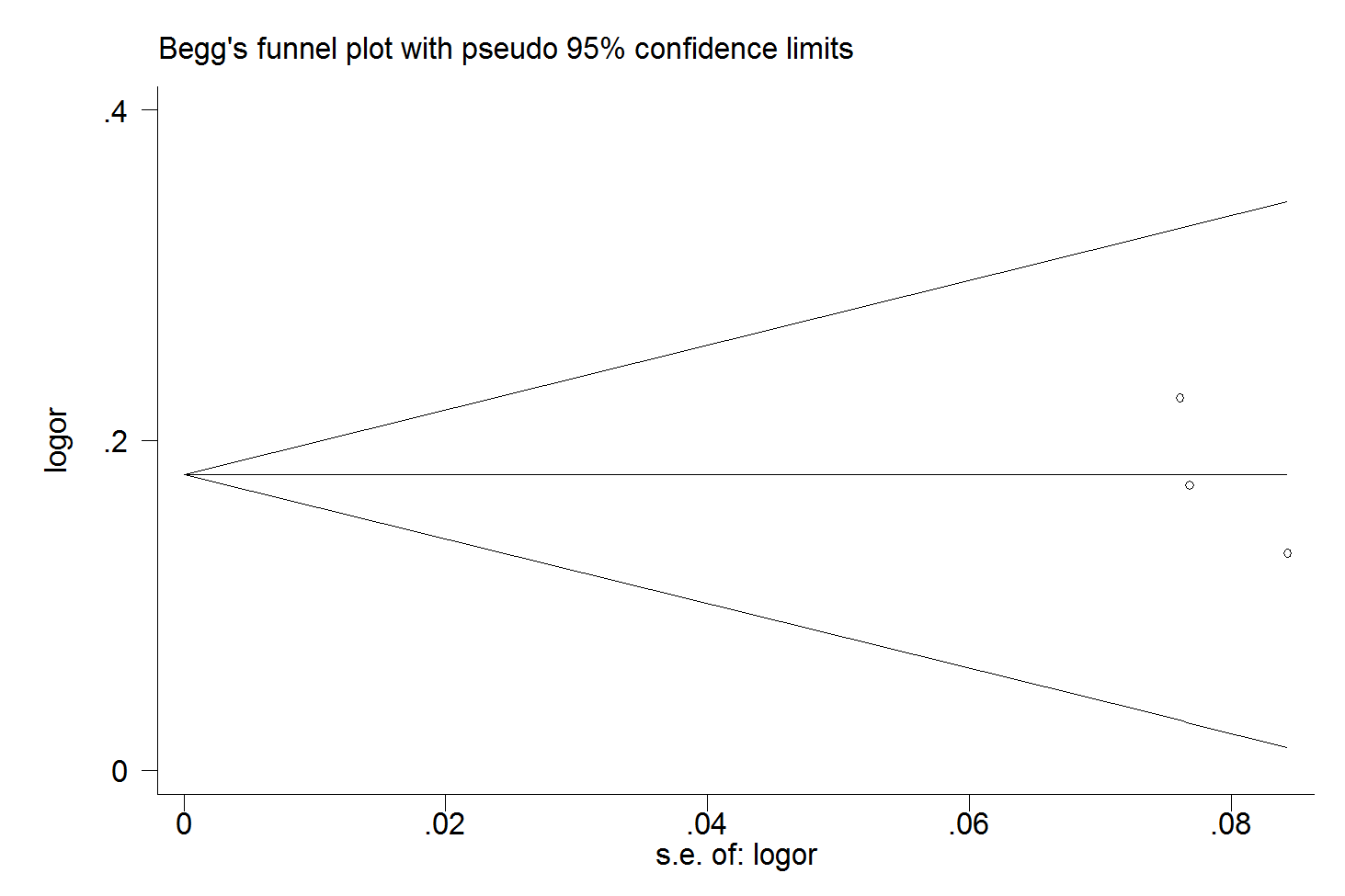

Supplement: Figure S16 — Begg's funnel plot of KCNQ1 rs2237892 polymorphism and gestational diabetes mellitus risk (Egger test, P = 0.34). (TIFF) [file pone.0045882.s016.tif]

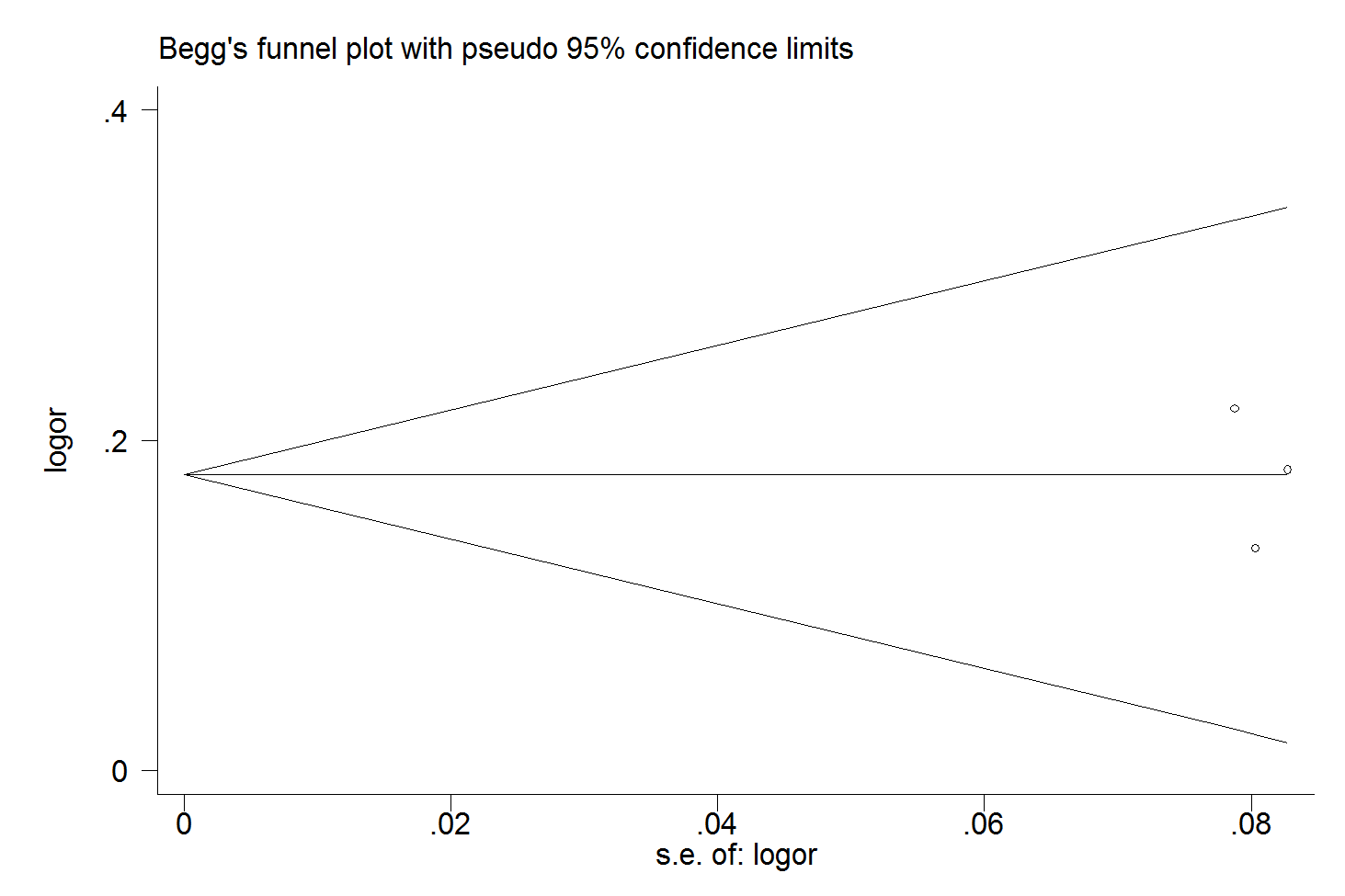

Supplement: Figure S17 — Begg's funnel plot of KCNQ1 rs2237895 polymorphism and gestational diabetes mellitus risk (Egger test, P = 0.77). (TIFF) [file pone.0045882.s017.tif]

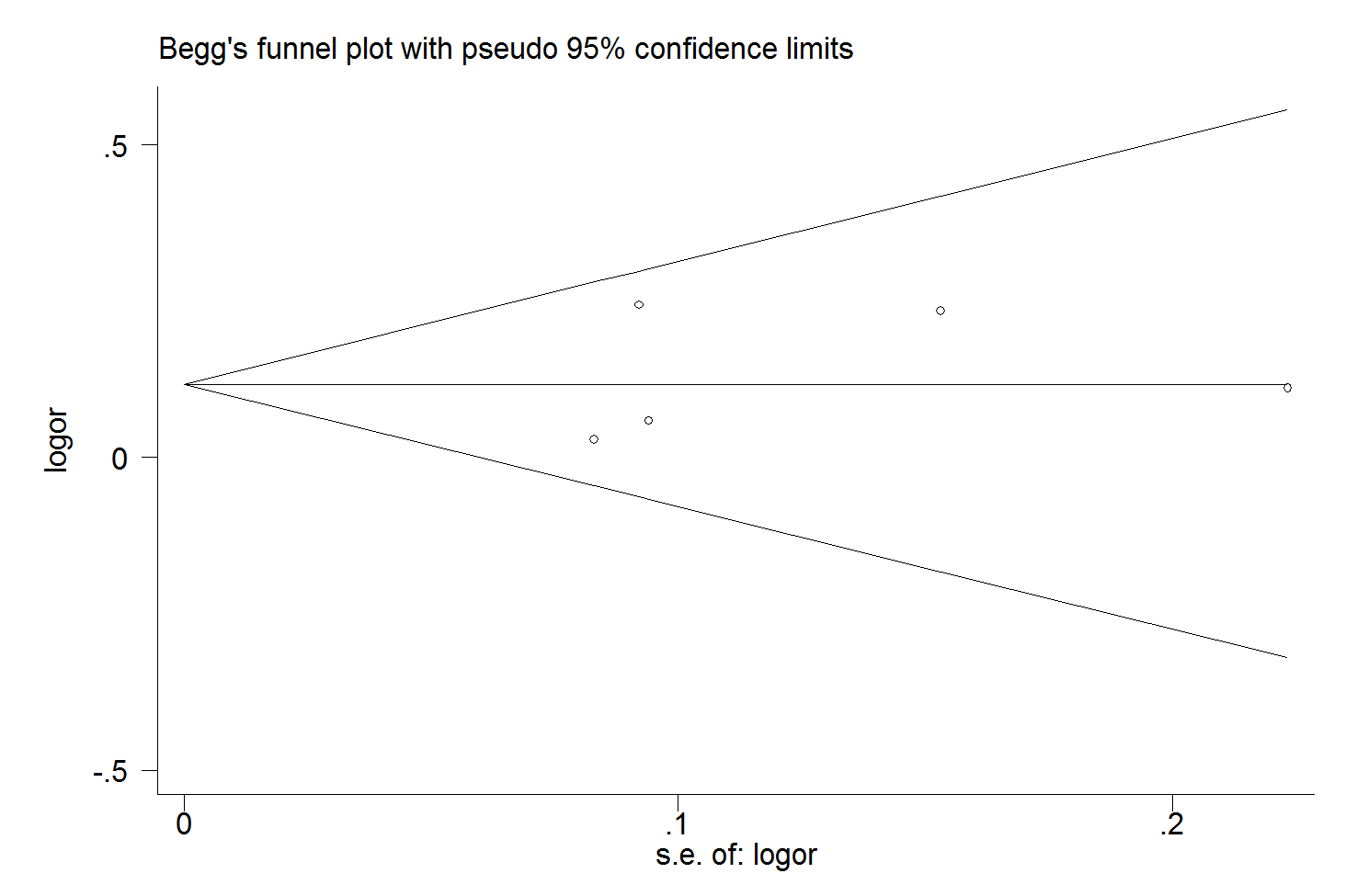

Supplement: Figure S18 — Begg's funnel plot of GCK rs4607517 polymorphism and gestational diabetes mellitus risk (Egger test, P = 0.65). (TIFF) [file pone.0045882.s018.tif]
